# Chemical Separation of Uranium and Precise Measurement of ^234^U/^238^U and ^235^U/^238^U Ratios in Soil Samples Using Multi Collector Inductively Coupled Plasma Mass Spectrometry

**DOI:** 10.3390/molecules25092138

**Published:** 2020-05-03

**Authors:** Nimelan Veerasamy, Asako Takamasa, Rajamanickam Murugan, Sharayu Kasar, Tatsuo Aono, Kazumasa Inoue, Masahiro Fukushi, Sarata Kumar Sahoo

**Affiliations:** 1 Environmental Radionuclide Research Group, National Institutes for Quantum and Radiological Science and Technology (QST), 4-9-1 Anagawa, Inage-Ku, Chiba 263-8555, Japan; nimelan.veerasamy@qst.go.jp (N.V.); atakamasa@gmail.com (A.T.); murugan.rajamanickam@qst.go.jp (R.M.); kasar.sharayu@qst.go.jp (S.K.); aono.tatsuo@qst.go.jp (T.A.); 2 Department of Radiological Sciences, Tokyo Metropolitan University, 7-2-10 Higashiogu, Arakawa-ku, Tokyo 116-8551, Japan; kzminoue@tmu.ac.jp (K.I.); fukushi@tmu.ac.jp (M.F.); 3 Energy Technology Unit, Japan Nus Co. (JANUS), 7-5-25 Nishi-Shinjuku, Shinjuku-ku, Tokyo 160-0023, Japan

**Keywords:** MC-ICP-MS, ^234^U/^238^U, ^235^U/^238^U, UTEVA resin, soil

## Abstract

A new chemical separation has been developed to isolate uranium (U) using two UTEVA columns to minimize iron and thorium interferences from high background area soil samples containing minerals like monazites and ilmenite. The separation method was successfully verified in some certified reference materials (CRMs), for example, JSd-2, JLk-1, JB-1 and JB-3. The same method was applied for purification of U in Fukushima soil samples affected by the Fukushima dai-ichi nuclear power station (FDNPS) accident. Precise and accurate measurement of ^234^U/^238^U and ^235^U/^238^U isotope ratios in chemically separated U were carried out using a multi-collector inductively coupled plasma mass spectrometer (MC-ICP-MS). In this mass spectrometric method, an array of two Faraday cups (10^11^ Ω, 10^12^ Ω resistor) and a Daly detector were simultaneously employed. The precision of U isotope ratios in an in-house standard was evaluated by replicate measurement. Relative standard deviation (RSD) of ^234^U/^238^U and ^235^U/^238^U were found to be 0.094% (2σ) and 0.590% (2σ), respectively. This method has been validated using a standard reference material SRM 4350B, sediment sample. The replicate measurements of ^234^U/^238^U in SRM shows 0.7% (RSD). This developed method is suitable for separation of U and its isotope ratio measurement in environmental samples.

## 1. Introduction

Uranium is the heaviest naturally occurring element on earth and it has three naturally occurring radioisotopes, ^234^U, ^235^U and ^238^U with relative isotopic abundances of 0.0055%, 0.72% and 99.27%, respectively [1]. A trace amount of ^236^U can occur naturally from neutron capture processes in uranium ores [2]. The accurate measurement of the isotopic composition of uranium will be useful to understand the variety of problems in the field of geology, hydrology, environmental sciences, nuclear safeguards and nuclear forensics [3,4]. Uranium isotope ratios vary in natural samples due to various physical, chemical, or even biological processes, including mass fractionation, redox transitions, radioactive decay, radioactive disequilibrium, alpha recoil, and neutron capture [5]. Significant natural variability of more than 0.03% in the ^235^U/^238^U ratio for a range of natural materials has been reported [6,7]. This variation is due to the different redox states and nuclear field shift effects. Large variations of ^234^U/^238^U in the environment (> 10%) can be observed mainly due to the alpha recoil effect. The disequilibrium of uranium can serve as a natural isotopic tracer for proving exposure pathways. Therefore, it is very essential to measure uranium isotopic composition in environmental samples, which requires high sensitivity to detect the smallest amount of minor isotopes (^236^U) and high accuracy to differentiate between small artificial components from natural uranium samples [8,9].

Over the past few decades, there is an increasing trend for the measurement of U isotope ratios using different types of inductively coupled plasma mass spectrometry (ICP-MS) such as quadrupole mass spectrometry (ICP-QMS), sector field-ICP-MS (SF-ICP-MS) and multi-collector ICP-MS. These techniques have proved to be extremely efficient and sensitive analytical mass spectrometric methods in ultra-trace analysis as well as precise and accurate determination of isotopic ratios of long-lived radionuclides in environmental samples [10,11,12]. An ongoing goal in environmental sciences is to develop a robust analytical method to provide useful data from a minimum amount of sample [13]. An enhanced sample introduction system interfaced with MC-ICP-MS has proved to be a powerful tool for high precision measurement of isotopic composition of long-lived radionuclides, leading to a wide array of new applications in the field of isotope geochemistry [14,15]. MC-ICP-MS has afforded simultaneous detection of ions with a multi-collector detector improved precision owing to high absolute sensitivity compared to quadrupole based ICP-MS and higher sample throughput compared to a thermal ionization mass spectrometer (TIMS). The MC-ICP-MS technique could determine accurately U isotope ratios at the femtogram level.

Precise measurements of isotope ratios in the MC-ICP-MS technique is significantly affected by spectral interferences arising from isobaric interferences, instrumental mass bias, or sample matrix effects due to the presence of other elements. Therefore, it is necessary to isolate U from the sample matrix to avoid any interferences. Adriaens et al. have separated U and Th in soil samples using a combination of anion exchange chromatography resin (AER, AG1X8, Bio-Rad, USA) and extraction chromatography resin (UTEVA, Eichrom Technologies, Inc., USA) [16]. This method purified U from Fe, Pb, and Bi and was a choice for environmental samples compared to any commercial strong anion resin separation. Later, Croudace et al. separated U and Pu in borate fusion soils using a combination of AER and UTEVA resin and measured U isotope ratios by (TIMS) [17]. In the early 2000′s Yokoyama et al. used for the first time two extraction chromatographic resins, such as UTEVA and TEVA (Eichrom Technologies, Inc., USA), to separate Th and U in silicate rocks [18]. Sahoo et al. (2004) employed AER and UTEVA resin for separation of U and its isotope ratio measurement in reference materials, Chernobyl soil samples and U ore. There were organic residues observed in the U fraction collected from UTEVA column. Therefore, a second AER column was introduced for further purification of U prior to measurement of isotope ratios using TIMS [3,9]. Mishra et al. (2014) separated U from Fukushima contaminated samples, which are rich in Fe concentration with high ^137^Cs activity concentration using a combination of AER and UTEVA resins [19]. Mishra et al. (2017) used a single UTEVA resin column to separate U from ore samples and measured U isotope ratios using TIMS [6].

The aim of our study is to develop a chemical separation method for U and its isotope ratios measurement using MC-ICP-MS from soil samples in high background radiation area (HBRA), Odisha, India, as well as Fukushima soil samples affected by the Fukushima dai-ichi nuclear power station (FDNPS), Japan, accident. HBRA soil samples are rich in monazite and ilmenite. Monazite is rare-earth phosphate mineral with appreciable substitution of thorium for rare earth and silicon for phosphorus. It is dominated by Th, U, Ce, La and Nd. Whereas ilmenite is a black iron-titanium oxide with a chemical composition of FeTiO_3_ [20]. The average dose rate of HBRA Odisha is 650 nGy h^−1^ compared to much higher than worldwide average value [21]. The enhancement of natural radioactivity is due to high concentration Th. In the case of Fukushima soil, Fe_2_O_3_ concentration ranges from 3 to 8 wt% with high ^137^Cs activity (~ 4500 Bq g^−1^) [22]. The objective of this study is as follows:

(1) ICP-MS was used for the determination of uranium concentration in one standard reference material (SRM) 4350B, four certified reference materials (CRM), such as JSd-2, JLk-1, JB-1 and JB-3, three HBRA and two Fukushima soil samples.

(2) A rapid chemical separation of U was developed using two UTEVA columns for high background area soil samples containing minerals like monazites (high concentration of U and Th) and ilmenite (high concentration of Ti) as well as high Fe content Fukushima soil.

(3) Precise measurement of ^234^U/^238^U and ^235^U/^238^U ratios were carried out using a MC-ICP-MS using two Faraday cups (10^11^, 10^12^ Ω resistor) and a Daly detector simultaneously.

## 2. Results and Discussion

### 2.1. Analytical Validation of U Measurement Using ICP-MS

A quadrupole ICP-MS was used for the measurement of stable isotopes of U, Th, La, Ce and Nd in reference materials and soil samples, which yielded detection limits of 0.01 μg/L. The ICP-MS detection limit was calculated as three times the standard deviation of the calibration blank measurements (1:1 *v*/*v* HNO_3_: MilliQ water, *n* = 10). The detailed parameters for data acquisition and optimization are reported elsewhere [23]. Isotope dilution internal standardization (ID-IS) mass spectrometry technique is one of the most accurate methods and current “state-of-art” in isotope ratio measurements. Multi-elemental plasma standard solutions and Rh as an internal standard were used to estimate intensity ratios (I) of the abundant stable isotopes of all elements of interest. Similarly, intensities of the respective isotopes were measured for samples spiked with an internal standard. From the comparison between standard and sample intensity ratios, total concentration of that element in the sample was calculated based on its natural abundance. To check the accuracy and validate the analytical procedure of U, one standard reference material SRM 4350B (River sediments) and four CRMs, for example, JSd-2 (Japan stream sediment), JLk-1 (Lake sediment), JB-1 and JB-3 (Japan basalts) supplied by the Geological Survey of Japan, were used. The relative standard deviation (RSD %) of U in reference samples were evaluated by triplicate analysis using ICP-MS and are given in Table 1. The concentration of U in reference materials varied from 0.46 to 3.77 μg g^−1^.

### 2.2. Measurement of U and Some Selected Elements in Soil Samples Using ICP-MS and X-ray Fluorescence Spectrometer

Major elements, for example, TiO_2_, Fe_2_O_3_, and P_2_O_5_ were characterized using an X-ray fluorescence spectrometer (XRF). The concentration of U, Th, La, Ce, Nd, TiO_2_, Fe_2_O_3_, and P_2_O_5_ in HBRA, Odisha and Fukushima soil samples are given in Table 2. The concentration of U and Th in HBRA soil ranged from 13 to 30 μg g^−1^ and 497 to 634 μg g^−1^, respectively, whereas Fukushima soils ranged from 1 to 2 μg g^−1^, and 9 to 11 μg g^−1^, respectively. The average Th/U ratio in Fukushima and HBRA soils are 4.5 and 32, respectively. The average continental crustal value of Th/U is 3.8 [24]. The ratio of Th/U reflects that the Fukushima soil derived from the granitic sources [25]. The HBRA samples are not only enriched in U and Th concentration but also have a high concentration of Fe, Ti, P, La, Ce and Nd due to the presence of monazites, ilmenite and rutile [26]. The values for Fe_2_O_3_ in HBRA soils varied from 8.2% to 12% whereas Fukushima soils were 4.9% and 6.3%. The concentration of U, Th, Fe_2_O_3_, TiO_2_ and P_2_O_5_ in HBRA and Fukushima soil samples are higher than the average upper continental crust [24]. Presence of high Fe content in both HBRA and Fukushima soil samples causes matrix effects during isotope ratio measurement of U by MC-ICP-MS. Therefore, it is strongly required to develop a clean chemical separation method to minimize contamination of Fe, Th and other elements from the soil matrix.

### 2.3. Analytical Chemical Separation of Uranium from a Synthetic Mixture and Samples

**Figure 1:**
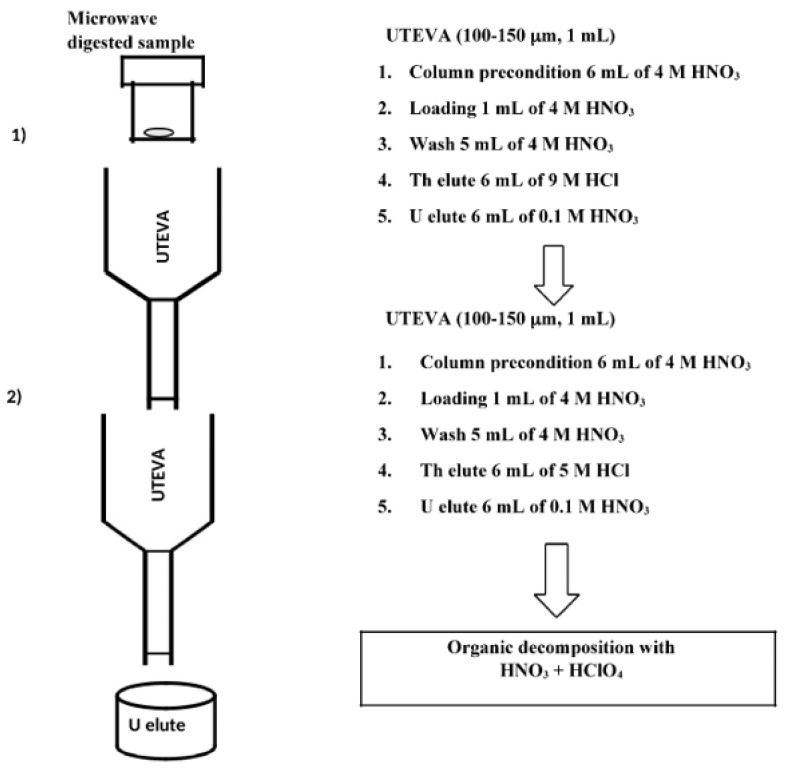
The chemical separation scheme of U using two UTEVA resin column.

A new separation method was developed to separate U from the soil matrix using two UTEVA extraction chromatographic resin columns. A synthetic mixture was prepared by mixing 1 mL of XSTC-1 (La, Ce, Pr, Nd, Sm, Eu, Gd, Tb, Dy, Ho, Er, Tm, Yb, Lu, Y; 10 μg mL^−1^), 1 mL of XSTC-13 (Th, Ag, Al, As, Ba, Be, Bi, Ca, Cd, Co, Cr, Cs, Cu, Fe, Ga, In, K, Li, Mg, Mn, Na, Ni, Pb, Rb, Sr, Tl, V, Zn, U, Hg; 10 μg mL^−1^) and 10 mL of Fe (1000 μg mL^−1^) for UTEVA extraction chromatographic resin experiment. The synthetic mixture of loading solution was prepared in the ratio of U and Fe as 1:1000. The UTEVA resin was precleaned by soaking overnight in 6 M HNO_3_ and then washed multiple times with Milli Q water. Finally, the precleaned UTEVA resin was stored in 0.5 M HNO_3_. The UTEVA column was prepared with 1 mL of precleaned UTEVA resin and preconditioned with 4 M HNO_3_. The schematic chemical separation for U using two UTEVA resin columns is given in Figure 1.

**Figure 2:**
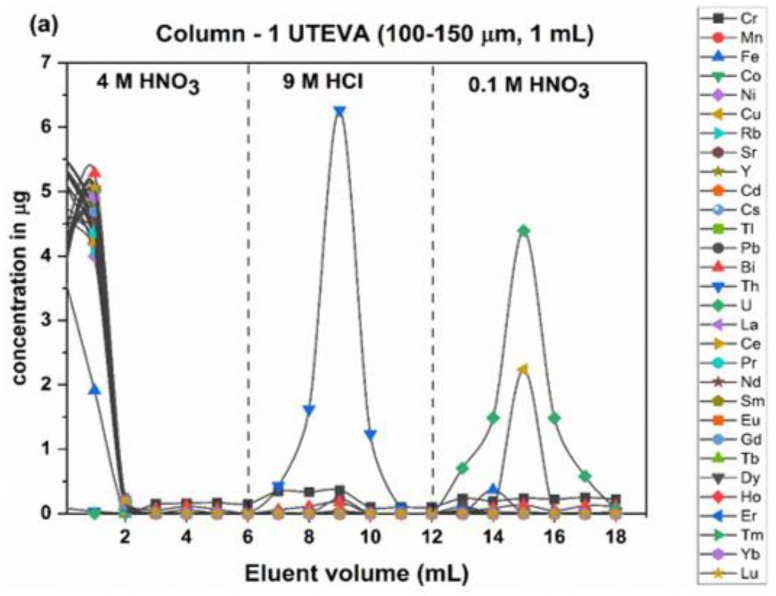

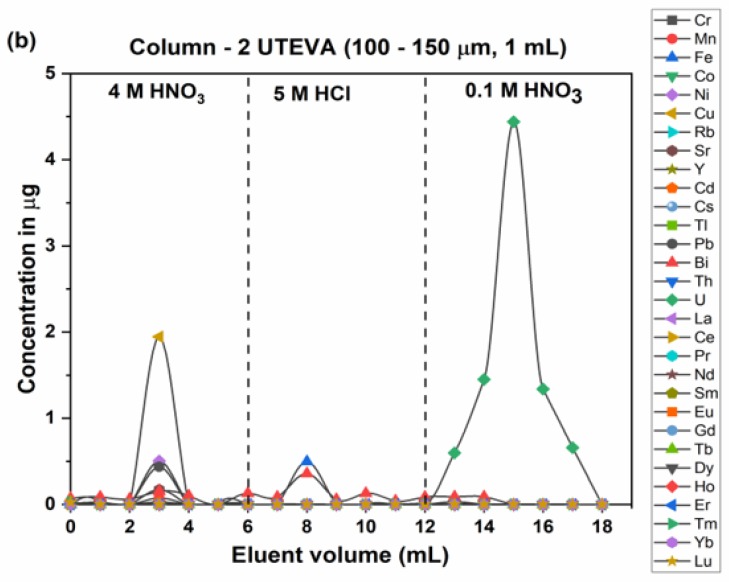
(**a**). Elution curves of U separation using fist UTEVA columns; (**b**). Elution curves of U separation using second UTEVA column.

The loading solution was conditioned to 4 M HNO_3_ and passed through the UTEVA column. The alkali metals (Li, Na, K, Rb and Cs), alkaline earth metals (Be, Mg, Ca, Sr and Ba), heavy metals (V, Cr, Mn, Fe, Co, Ni, Cu, Zn, Ga, As, Ag, Cd, In, Hf, Tl, Pb, Bi and Hg) and rare earth elements (La, Ce, Pr, Nd, Sm, Eu, Gd, Tb, Dy, Ho, Er, Tm, Yb, Lu and Y) were washed off with 6 mL of 4 M HNO_3_ from the column. Later, Th was eluted with 6 mL of 9 M HCl. Recovery of Th was about 90% in this fraction. Finally, U was eluted by passing 6 mL 0.1 M HNO_3_ onto the column. Recovery of U was about 90%. The elution profiles of U chemical separation from the synthetic mixture of elements contained in XSTC-1, XSTC-13 and Fe solution are given in Figure 2a. However, complete purification of U was not possible due to the presence of Fe and Cu. Therefore, another UTEVA column was used for further purification of U.

Figure 2b shows the chemical separation of U in the second column. The second UTEVA resin column was prepared the same as the first UTEVA column. The final fraction containing U with other elements in the first column was conditioned to 4 M HNO_3_ for the second column. Then it was loaded into the second column and passed 6 mL of 4 M HNO_3_. Traces of Cu, Ni and Pb were removed in 4 M HNO_3_ fraction. Instead of 9 M HCl, 5 M HCl was passed in the second UTEVA resin column that eluted Bi and Fe completely. Finally, U was eluted with 0.1 M HNO_3_, without any traces of other metals. In order to estimate U recovery in fractions before and after separation with two UTEVA columns, ^238^U content was analyzed in all samples. The separation recovery (%) for all elements was calculated using the formula, (100*C_A_/C_B_); where C_A_ and C_B_ is the concentration of an element after and before column separation, respectively. The cumulative U recovery of U using two UTEVA columns is about 85%.

This two UTEVA column separation method was used for separation of U in reference materials, HBRA and Fukushima soil samples. The recovery of U ranged from 80% to 95%. The total time required for this experiment including sample preparation, decomposition and chemical separation is three days. This method avoids high concentration of HNO_3_ acid and uses less volume of acids; thus proved to be an efficient chemical separation of U over conventional anion exchange or solvent extraction separation. This present method is rapid compared to the previous method followed in our laboratory [3,27]. However, there was more organic residue in the final U fraction while using two UTEVA extraction chromatographic resin columns. In order to overcome organic matters in the final fraction, a mixture of HNO_3_ and HClO_4_ was used to decompose organics. This step is necessary to ensure the beam stability in MC-ICP-MS measurement. The recovery of U concentration was quantified using ICP-MS. After organic decomposition, 50 ng mL^−1^ of U solution were prepared with 2% HNO_3_ for the isotope ratio measurement using MC-ICP-MS. In this study, U separated from JB-1 (Basalt) was used as an in-house standard throughout U isotope ratio measurement using MC-ICP-MS.

### 2.4. Optimization of MC-ICP-MS for U Isotope Ratios Measurement

The routine optimization of MC-ICP-MS was performed by adjusting the torch position, nebulizer gas flow, ion lens positions, and high-tension (HT) voltages. This resulted in the most optimal and stable beam intensity for the ^238^U beam collected in a Faraday cup detector (L2) using an in-house uranium solution and reduced the background noise. Table 3 lists typical operating and measurement conditions for uranium analysis.

The acid concentration in standards and samples was maintained at 2% HNO_3_ during U isotope ratio measurements. U isotope ratio measurements were carried out in both wet plasma and dry plasma mode. In wet plasma mode, the measurement was performed using 50 ng mL^−1^ U concentration solution. Sample solutions were introduced into the plasma through a micromist nebulizer with an aspiration rate of 200 µL min^−1^. After each measurement, a washout was performed with 2% HNO_3_ for 15 min. The isotope ratio measurement comprises of 15 blocks of 10 cycles with an 8 s integration time. One block consists of 10 cycles (isotope ratios). The instrumental background (zero measurement) was measured by introducing 2% HNO_3_ before sample measurement.

In dry plasma mode, Aridus-3 desolvating nebulizer (Teledyne CETAC Technologies, USA) was used where 20 ng mL^−1^ of U concentration solution was aspirated at a rate of 100 µL min^−1^. The Ar sweep gas flow rate was typically 4.25 L min^−1^ with a nebulizer gas flow rate of 0.9 L min^−1^. No additional N_2_ gas was used for the measurement. The other instrumental operating settings are identical to wet plasma measurements. The washout was approximately 30 min using 2% HNO_3_. U in-house standard with concentrations of 20 and 50 ng mL^−1^ was measured before and after the sample measurement, respectively. The concentrations of U consumed in one measurement were approximately 40 and 200 ng for dry and wet plasma mode, respectively.

**Figure 3:**
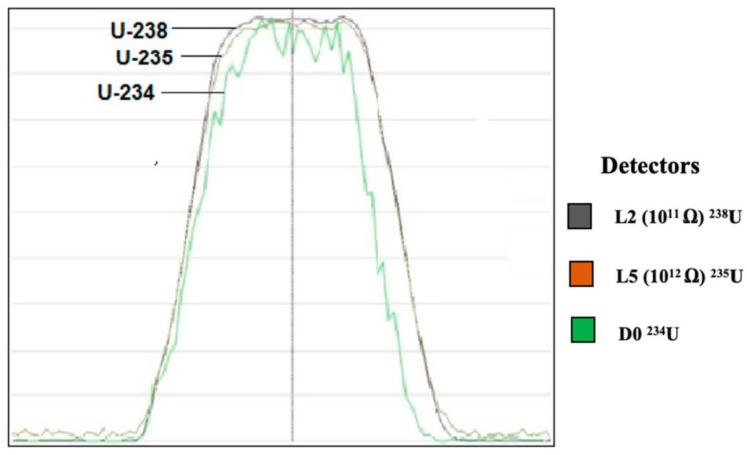
Multi scan of uranium isotopes in 50 ng mL^−1^ ((Two Faraday cups; L2—^238^U and L5—^235^U) and (Daly detector; D0—^234^U)).

The U concentration in the sample was in µg mL^−1^ range. Therefore, wet plasma mode has been employed for U isotope (^234^U/^238^U and ^235^U/^238^U) ratio measurement. The maximum intensity of U isotopes in 50 ng mL^−1^ were 3.6 V, 0.023 V and 11500 cps for ^238^U, ^235^U, and ^234^U, respectively. ^234^U/^238^U and ^235^U/^238^U isotope ratios were calculated as the mean ratio of 150 replicates with an outlier test that was implemented in Nu plasma 3D instrument software (Nu Instruments Calculation Editor-NICE). The values that deviated from the mean by more than two-sigma were considered as outliers. The multi scan of U isotopes peak shape and peak coincidence are shown in Figure 3. The exponential fractionation law has been used for internal mass bias correction.

An Excel spreadsheet was used for assembling data outputs and further external correction was performed by the standard sample bracketing technique [28] using the equation given below.

(1) $$K_{Std,\;i/j}=\frac{R_{Std,\;i/j}}{r_{Std,i/j}}$$

(2) $$R_{Sample,i/j}=K_{Std,i/j}\times r_{Sample,i/j}$$

where, *R_Std,i/j_* is the certified value of standard, *r_Std,i/j_* is a measured value. *K_Std,i/j_* is a conversion factor, *R_sample,i/j_* is the corrected value and *r_Sample,i/j_* is a measurement value. The conversion factor of the standard has been calculated using Equation (1) and that has been applied in Equation (2) to calculate the true ratio of the analyte in the sample. Comparable corrections were applied to the respective isotope ratios in samples. The uncertainty of mass bias correction was estimated by using the external standard sample bracketing technique at a 95% confidence interval [28]. The molecular interference from ^238^UH^+^ was measured and corrected online using exponential law during the measurement. However, it was negligible. The peak tailing, Faraday cup amplifier gain, and baseline associated uncertainties were very small and negligible in our case.

### 2.5. Measurement of U Isotope Ratios for in-House Standard Using MC-ICP-MS

**Figure 4:**
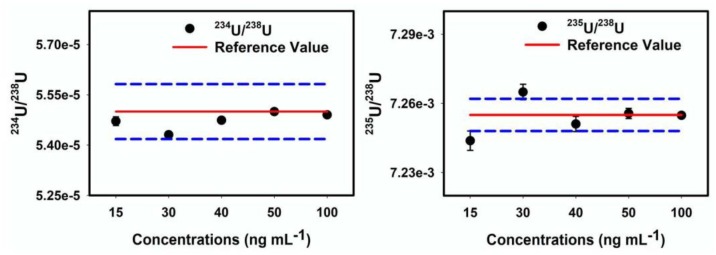
^234^U/^238^U and ^235^U/^238^U isotope ratio measured on different concentrations of uranium in ‘in-house standard’. The blue dotted line indicated twice the standard deviation (2SD) of the measurement.

**Figure 5:**
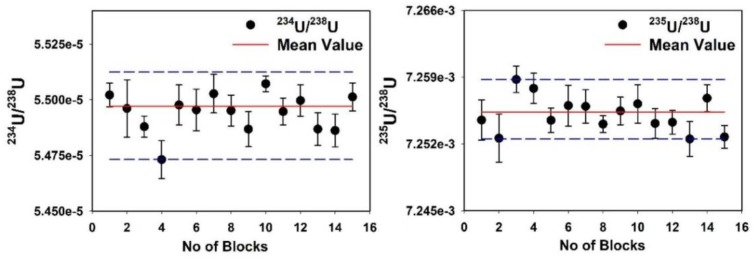
Stability of U isotope ratios measured by MC-ICP-MS. The blue dotted line indicated twice the standard error (2SE) of the measurement.

It is important to optimize the concentration of U for the MC-ICP-MS measurements in wet plasma mode because it helps to improve the stable performance of U isotope ratio measurement. To establish the working standard, different concentrations of 15, 30, 40, 50 and 100 ng mL^−1^ U solutions were prepared from purified U fraction of the in-house standard. Figure 4 illustrates that the most optimal U concentration for isotope ratio measurement was 50 ng mL^−1^. The 50 ng mL^−1^ U concentration has a relative standard deviation (RSD %) of 0.027% (2σ) and 0.161% (2σ) for ^234^U/^238^U and ^235^U/^238^U isotope ratios, respectively. The stability of U isotope ratios measured in a single measurement of the in-house standard is shown in Figure 5. The background intensity of U isotopes measured in 2% HNO_3_ are *^238^U* < 0.003 V, *^235^U* < 0.001 V and *^234^U* < 1 cps.

### 2.6. Validation of U Isotope Ratio Measurement Using MC-ICP-MS

To validate our U isotope ratio measurement using MC-ICP-MS, the ISO Guide to the Expression of Uncertainty in Measurement (GUM) [29] and the Eurachem/CITAC Guide “Quantifying Uncertainty in Analytical Measurement” [30] has been followed. The details of the estimation of accuracy and precision for the method used in the present study are given here.

#### 2.6.1. Accuracy and Precision for In-House Standard (JB-1) Using MC-ICP-MS

Isotope ratios of the in-house standard were measured on seven different dates from February 2020 to March 2020 to find a long-term analytical reproducibility of the instrument (Table 4). The reproducibility obtained for ^234^U/^238^U and ^235^U/^238^U isotope ratios are 0.094% (2σ) and 0.590% (2σ) relative standard deviation, respectively.

The significant difference between the measured mean U isotope ratio and seven different dates of U isotope ratios of in-house standard results was calculated considering their uncertainties. In this process, the absolute difference (AD) for the measured values was compared with the uncertainty of the absolute difference (U_AD_) [29]. The AD and U_AD_ were calculated by the following equations.

(3) AD=|R−M|

(4) $$U_{\mathrm{AD}}=\sqrt{{\left(U_{R}\right)}^{2}+{\left(U_{M}\right)}^{2}}$$

where *R* is the U isotope ratios for seven different dates, and *M* is the mean of all U isotope ratios for the in-house standard. *U_R_* and *U_M_* are the uncertainties on the U isotope ratios for seven different dates and the mean U isotope ratio for the in-house standard, respectively. If *AD* ≤ 1.96U_AD_, then the U isotope ratios measured on seven different dates and the mean U isotope ratio for in-house standard in this study are equal as well as accepted as ‘precise’ with a confidence interval of 95%. All the U isotope ratios measured on seven different dates showed AD values less than 1.96U_AD_ and were within the error range. No larger isotopic variations were observed among the seven different dates and the mean U isotope ratio for the in-house standard. The mean of seven different measurements for the in-house standard was compared with the natural U isotope ratios for the accuracy. They are found to be in good agreement. The calculated relative bias (RB) for ^234^U/^238^U and ^235^U/^238^U isotope ratios of the in-house standard was less than 10% of the maximum acceptable relative bias (MARB) [30]. The internal precision obtained for ^234^U/^238^U and ^235^U/^238^U isotope ratios was 3 and 0.01 ppm, respectively. From this, it is illustrated that the U isotope ratio measurement using MC-ICP-MS is accurate and precise.

#### 2.6.2. Validation of ^234^U/^238^U Ratio for NIST SRM 4350B

**Figure 6:**
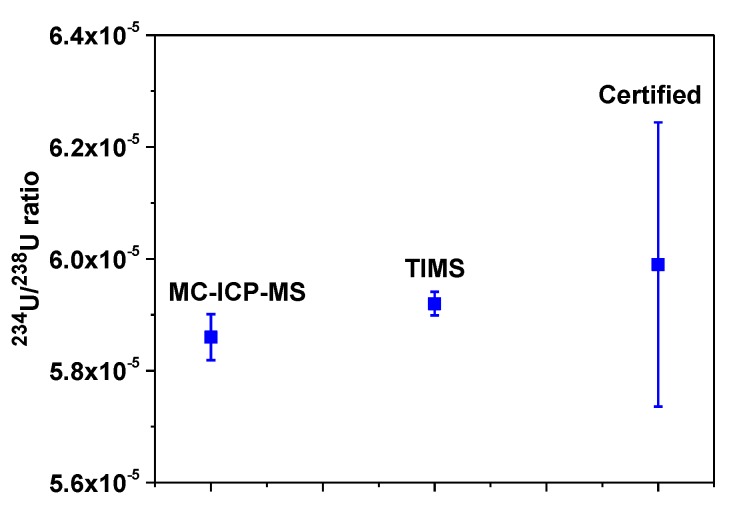
Comparison between measured values and certified values of ^234^U/^238^U ratio for National Institute of Standard and Technology (NIST) 4350B.

The validation of the ^234^U/^238^U ratio measurement was carried out using NIST (National Institute of Standard and Technology, USA) SRM 4350B. The certified ratio of ^234^U/^238^U is 5.99 × 10^−5^ (± 2.54 × 10^−6^) [31]. The measured ratio in the present work is 5.86 × 10^−5^ (± 4.10 × 10^−7^). The measured ratios for other CRMs and samples are reported in Table 5. The absolute difference (AD) between mean measured ratio and the certified ratio is less than twice the uncertainty of U_AD_. This supports the good performance of our method since there is no significant difference between the measured ratio and the certified ratio. In order to evaluate the analytical accuracy of our measurement, RB has been calculated [32]. The relative bias for SRM 4350B is (−2.17%). The MARB for all U isotope ratios is set to 10%. If the |RB| < MARB then the result could be ‘Acceptable’ for accuracy. Hence, our measured ratio is ‘Acceptable’, implying accuracy of the measurement. The reproducibility in this measurement has been validated from RSD (%). In this case, RSD% of the measured value is ~0.70 which is lower than the certified value of ~4.24. Based on the above criteria, the accuracy and precision of the U isotope ratio measurement using MC-ICP-MS in our study confirmed to be valid. The ratios of ^234^U/^238^U by TIMS, MC-ICP-MS and radiometry (certified value) are plotted in Figure 6. The difference in the relative standard deviation percentages for these three methods incorporates the precision of the measurement.

### 2.7. Measurement of ^234^U/^238^U and ^235^U/^238^U Isotope Ratios in Reference Materials and Environmental Samples

The measured results of ^234^U/^238^U and ^235^U/^238^U isotope ratios for reference materials, HBRA and Fukushima soil samples are given in Table 5. ^235^U/^238^U ratio in reference materials, HBRA, and Fukushima soil samples has been compared to the well known “normal terrestrial ratio” 0.00725 [3]. The ratios of ^235^U/^238^U in both HBRA, and Fukushima soil samples do not show any enrichment of ^235^U/^238^U. The activity ratios of ^234^U/^238^U were calculated from the isotopic abundance ratio with decay constant of ^234^U and ^238^U. ^234^U/^238^U activity ratio of CRMs, HBRA and Fukushima soils are ranging from 0.97 to 1.05, which exhibits a secular equilibrium between ^234^U and ^238^U. There was a little heterogeneity noticed in the case of the reference materials, for example, sediments and the Fuk–2 sample. However, such variations are within analytical errors.

## 3. Materials and Methods

### 3.1. Reagents

All chemical procedures and measurements were performed under clean room conditions. Ultrapure water with specific resistance > 18.3 MΩ cm obtained from a Direct-Q 3 UV water purification system (Millipore, Moisheim, France) and ultrapure analytical grade acids HNO_3_, HClO_4_ and HF (Tama chemicals, Kawasaki, Japan) were used throughout analytical procedures. Multi-element plasma standard solutions XSTC-1 (La, Ce, Pr, Nd, Sm, Eu, Gd, Tb, Dy, Ho, Er, Tm, Yb, Lu, Y; 10 μg mL^−1^), XSTC-13 (Th, Ag, Al, As, Ba, Be, Bi, Ca, Cd, Co, Cr, Cs, Cu, Fe, Ga, In, K, Li, Mg, Mn, Na, Ni, Pb, Rb, Sr, Tl, V, Zn, U, Hg; 10 μg mL^−1^) (Spex CertiPrep, Inc., Meteuchen, NJ, USA) and Fe (1000 μg mL^−1^; Wako Pure Chemical Industries, Ltd., Osaka, Japan) were used to check chemical separation of U and calibration curve for ICP-MS measurement.

### 3.2. Samples

Standard reference material SRM 4350B (river sediment) supplied by the National Institute of Standard and Technology (NIST, Gaithersburg, MD, USA) and four certified RMs, for example, JSd-2 (Japan stream sediment), JLk-1 (Japan lake sediment), JB-1 and JB-3 (Japan basalt) supplied by Geological Survey of Japan (GSJ, Tsukuba, Japan) were used. Two different types of soils, for example, HBRA Odisha samples (HBRA-1, 2 and 3) collected from eastern coastal region of Odisha state, Chhatrapur, India, and Fukushima soils (Fuk-1and 2) collected within 30 km from the Fukushima dai-ichi nuclear power station (FDNPS), Namiemachi, Japan.

### 3.3. Sample Preparation

The surface soil samples were collected from HBRA, Odisha, India, and Fukushima, Japan. Approximately 2 kg of samples were collected and brought to the laboratory; a detailed sample collection method is mentioned elsewhere [23]. The samples were dried at 110 °C for 24 h to remove moisture. Then after removing the extraneous materials, such as plant roots, dead leaves and gravel, the samples were sieved using a 2 mm sieve and further homogenized and pulverized to less than 150 μm grain size. About 250 mg of homogenized samples were ashed in a muffle furnace (KDF-S70, Tokyo, Japan) at 600 °C for 6 h to decompose organic matter. After ashing, the samples were digested with a mixture of HNO_3_, HF and HClO_4_ in a closed PTFE pressure vessel using a microwave digestion system (MLS 1200 mega, Milestones, Sorisole, Italy). The sample solution was transferred into Teflon beakers and evaporated to dryness on a hot plate. Finally, the residue was dissolved with 3% HNO_3_ to yield the sample solution for ICP-MS measurement. An internal standard, Rh, was spiked into each sample to correct the signal attenuation due to the presence of various constituents in the sample (known as the “matrix effect”) as well as for possible changes in instrumental parameters.

### 3.4. Extraction Chromatography Resin (UTEVA)

The commercial extraction chromatographic resin, UTEVA (100–150 μm) was purchased from Eichrom Technologies Inc., Lisle, IL, USA). UTEVA resin consisted of a neutral organophosphorus diamyl amylphosphonate extractant adsorbed onto an inert polyacrylamide support. MUROMAC polypropylene S size column (5 × 50 mm) was used for the extraction column separation of U.

### 3.5. Instrumentation

#### 3.5.1. XRF

The measurements of the major elements of the prepared bead samples were carried out using a wavelength dispersive XRF spectrometer (ZSX100e, Rigaku, Tokyo, Japan) equipped with an X-ray tube 4 kW Rh anode and ultrathin Rh end window. The detailed sample preparation procedure for XRF analysis has been mentioned elsewhere [27].

#### 3.5.2. ICP-MS

ICP-MS (Agilent-8800, Agilent Technology, Tokyo, Japan) was used to determine the U, Th, La, Ce and Nd concentration in reference materials and soil samples. It was used to find the recovery of U in the chemical separation.

#### 3.5.3. MC-ICP-MS

Uranium isotope ratios were measured employing a double-focusing MC-ICP-MS, Nu Plasma 3D (Nu Instruments Ltd., Wrexham, UK) established at National Institutes for Quantum and Radiological Sciences (QST), Fukushima, Japan. The mass spectrometer is equipped with fixed sixteen Faraday cups and five Daly detectors (Table 6). The Faraday cups of H1, L1, L2, L3, L4, L5 are equipped with switchable dual resistors of 10^11^ Ω and 10^12^ Ω resistors, H8, H7, H6, H5, H4, H3, H2, Ax Faraday cups with 10^11^ Ω resistor, and L6, L7 Faraday cups with 10^12^ Ω resistor. The Faraday cups with 10^11^ Ω and 10^12^ Ω resistors can measure a maximum signal intensity of 50 and 5 V, respectively. The 10^12^ Ω resistor provides 10 times higher voltage compared to the 10^11^ Ω resistor for a given ion beam and Johnson Nyquist (JN) noise level of the resistor increases by a factor of $\sqrt{10}$. Theoretically, it could improve the signal to noise ratio by 3-fold. The application of the 10^12^ Ω resistor Faraday cup detectors are mainly used to measure low ion intensity below 0.1 V [33]. The Daly detectors are located at the lower side of the detector system, between L5 to L7 Faraday cup detectors. The application of a retardation filter for the Daly detectors helped to minimize the impact of the potential tailing effect of the larger isotope signal to the low abundance isotope signals. Daly detector can measure a maximum signal intensity of about 0.160 V (10 million counts per second). The Faraday gain calibrations were performed every day and before that measurement.

The two-collector configuration was used for uranium isotope ratio measurement using MC-ICP-MS. ^238^U and ^235^U ions were measured with Faraday cups using L2 (10^11^ Ω), L5 (10^12^ Ω) resistor, respectively, whereas ^234^U on the Daly detector. In the second configuration, the ^238^U was collected in an L3 (10^11^ Ω) Faraday cup and ^235^U was collected in a D0 Daly detector (Table 6). Using two different cup configurations, the drift in the relative gain between the Daly and Faraday cup detectors was estimated and the stability of low abundant uranium isotope signals were measured.

## 4. Conclusions

In this study, the concentration of U was measured using ICP-MS in certified reference materials and there was a good analytical agreement within an error of ± 10%. The newly developed two UTEVA extraction chromatography resin column resulted in clean chemical separation of U from the soil matrix. The method was applied to HBRA and Fukushima soils. Advantages of the two UTEVA resin method reduced the volume of acids and molarity of acids, which is eco-friendly. Isotope ratios of U were accurately measured using MC-ICP-MS from the chemically purified U solution. The internal precision obtained for ^234^U/^238^U and ^235^U/^238^U isotope ratios using MC-ICP-MS was 3 and 0.01 ppm, respectively. This method could be applicable to measure the ^234^U/^238^U ratio at the trace level in Fukushima ground water samples to detect any U disequilibrium studies in the future.

## Figures and Tables

**Table 1:** U concentration of reference materials measured using inductively coupled plasma mass spectrometry (ICP-MS).

Sample Name	Certified Value of U (μg g^−1^)	Measured Value of U (μg g^−1^) in Soil	RSD (%)
SRM 4350	2.51	2.50 ± 0.5	1.9
JSd-2	1.1	1.02 ± 0.01	3.8
JLk-1	3.83	3.77 ± 0.14	3.7
JB-1	1.67	1.65 ± 0.03	1.7
JB-3	0.48	0.46 ± 0.01	4.5

**Table 2:** Elemental concentration of high background radiation area (HBRA) and Fukushima samples.

Sample Name	GPS Co-Ordinates		D	La	Ce	Nd	Th	U	TiO_2_	Fe_2_O_3_	P_2_O_5_
			nGy h^−1^	μg g^−1^					wt%		
HBRA-1	N 19.323	E 84.960	572	18 ±1.3	2454 ± 2	826 ± 0.2	497 ± 0.4	16 ± 0.02	9.4	10.5	0.29
HBRA-2	N 19.258	E 84.905	1064	15.5 ± 2	2344 ± 7	783 ± 1	587 ± 1	13 ± 0.01	7.7	8.2	0.24
HBRA-3	N 19.347	E 84.946	1224	1127 ± 0.3	2317 ± 5	946 ± 0.1	634 ±0.1	30 ± 0.2	8.9	12	0.18
Fuk-1	N 37.552	E 140.918	7507	23.6 ± 0.3	45 ± 0.5	19.1 ± 0.3	11 ± 0.07	2.1± 0.01	0.7	6.3	0.09
Fuk-2	N 37.498	E 140.969	19562	17.6 ± 0.2	36 ± 0.6	14 ± 0.2	9.8 ± 0.1	2 ± 0.03	0.5	4.9	0.07
UCC				32	65	26	10	2.5	0.5	4.9	0.1

UCC—Upper continental crustal values [24]. Concentration of La, Ce, Nd, Th and U are expressed as μg g^−1^ whereas TiO_2_, Fe_2_O_3_ and P_2_O_5_ are wt%.

**Table 3:** Multi-collector inductively coupled plasma mass spectrometer (MC-ICP-MS) operating and measurement conditions.

RF Power	1300 W
Acceleration Potential (V)	6000
Sampler cone	Ni cone
Skimmer cone	Ni wide angle cone
Resolution	Low
Cool gas	13.4 L min^−1^
Auxiliary gas	0.90 L min^−1^
	**Wet Plasma**
Sample	Conventional Spray chamber
Nebulizer	Micromist, 200 µL min^−1^
Nebulizer gas	1.14 L min^−1^
Sweep Ar Gas	-
Cycles/Blocks	10 cycles/15 blocks
Sample Concentration	50 ng mL^−1^
Typical Sensitivity	50 V per µg mL^−1^
Washout time 2% HNO_3_	10–15 min
^238^U Beam intensity	2.6–3.6 V

**Table 4:** Replicate analyses of Uranium isotope ratio measurement of the in-house standard (JB-1).

Date	^234^U/^238^U	RSE%	^235^U/^238^U	RSE%
2020-Feb-27	0.0000553 (01)	0.253	0.0072550 (30)	0.045
2020-Feb-28	0.0000554 (01)	0.248	0.0072467 (27)	0.040
2020-Mar-02	0.0000553 (13)	0.240	0.0072557 (29)	0.040
2020-Mar-03	0.0000554 (02)	0.246	0.0072489 (26)	0.035
2020-Mar-09	0.0000547 (02)	0.231	0.0072567 (26)	0.039
2020-Mar-10	0.0000546 (02)	0.254	0.0072506 (28)	0.039
2020-Mar-11	0.0000546 (06)	0.249	0.0072566 (31)	0.046
Mean	0.0000551 (04)	0.246	0.0072529 (28)	0.045

Uncertainties are expressed in 2σ_m_.

**Table 5:** Uranium isotope ratios in reference materials and environmental samples using MC-ICP-MS.

Sample Name	^234^U/^238^U	^235^U/^238^U
SRM 4350B	0.0000586 (01)	0.0072527 (50)
JSd-2	0.0000566 (02)	0.0072597 (57)
JLk-1	0.0000587 (01)	0.0072499 (43)
JB-1	0.0000547 (02)	0.0072543 (30)
JB-3	0.0000551 (03)	0.0072560 (68)
HBRA-1	0.0000539 (04)	0.0072594 (05)
HBRA-2	0.0000546 (03)	0.0072500 (09)
HBRA-3	0.0000541 (04)	0.0072545 (69)
Fuk-1	0.0000552 (02)	0.0072523 (20)
Fuk-2	0.0000564 (04)	0.0072585 (19)

Uncertainties are expressed in 2σ_m_.

**Table 6:** Configuration of collectors in MC-ICP-MS.

Detectors	L7	D4	D3	D2	D1	L6	D0	L5	L4	L3	L2	L1	Ax	H1	H2	H3	H4	H5	H6	H7	H8
^1st^ Configuration							^234^U	^235^U			^238^U	^238^UH^+^									
2nd Configuration							^235^U			^238^U	^238^UH^+^										
Faraday Cup resistors	10^12^Ω					10^12^Ω		10^12^Ω	10^11^Ω	10^11^Ω	10^11^Ω	10^11^Ω	10^11^Ω	10^11^Ω	10^11^Ω	10^11^ Ω	10^11^Ω	10^11^Ω	10^11^Ω	10^11^Ω	10^11^Ω
